# Understanding solastalgia from a decolonised, Indigenist lens: a scoping review

**DOI:** 10.3389/fpubh.2023.1261247

**Published:** 2024-01-15

**Authors:** Kisani Upward, Kim Usher, Vicki Saunders, Myfanwy Maple

**Affiliations:** ^1^School of Health, University of New England, Armidale, NSW, Australia; ^2^Centre for Indigenous Health Equity Research, First Nations Academy, Central Queensland University, Townsville, QLD, Australia

**Keywords:** Aboriginal, Australia, solastalgia, Country, resilience

## Abstract

The objective of this review is to use a decolonised, Indigenist lens to understand the definition of solastalgia from an Aboriginal perspective, as a potential emotional response experienced by Aboriginal communities impacted by increasingly frequent natural disasters, such as bushfires. Initial search results revealed a lack of literature referencing solastalgia in an Aboriginal-specific context. Indigenist research methodologies such as Heuristic inquiry and Aboriginal Participatory Action Research (APAR) contributed to the identification of alternative terminologies, which led to the majority of the included publications referring to solastalgia-related concepts, with one included publication mentioning solastalgia specifically. These methods were consequently used to synthesize data, confirm results and contribute to final discussions. Lastly, our results determined that at this stage there is insufficient evidence to conclusively suggest that Aboriginal Peoples in a general sense, experience solastalgia. Acknowledging the ethical dilemma and potential harm of generalising personal philosophies and experiences based on Culture. Thereby, signify the need for further research in this space and in particular, from a decolonised, Indigenist perspective.

**Preface:**

In the context of this review, we as co-authors are mindful of and respect the tensions or politics associated with proclaiming or discussing the identities of Australia's First Peoples. Therefore, in the context of this review, the often preferred term *Aboriginal* refers to the traditional owners of Australia. Where possible, traditional place/tribe names are written to acknowledge the ownership and origins of the information referenced within this review. Furthermore, we wish to acknowledge the storeys and traditional knowledge shared by the authors of the studies referenced within this review. These words of Country and Kin have contributed to the development and conceptualisation of this literature review, and we wish to pay our respects and appreciation.

## 1 Foreword

Aboriginal peoples within Australia have experienced extreme adversity since the dawn of colonial invasion. Since this time, Australia has witnessed rapid industrial growth and widespread development, often resulting in detrimental changes to the environment. The ecosystem has, in many ways and many places, suffered irreversible damage and experienced great biological losses as a result of the environmental disruption (1). Additionally, studies suggest that ecosystems of settled regions have deteriorated as a result of the displacement of Aboriginal Peoples, who traditionally tended to the land (1). Consequently, the impact of disconnection from Country [a term often used by Aboriginal peoples to describe the lands, waterways, and seas to which they are connected (2)] has disrupted and, in some cases, ceased the practise of intergenerational transference of cultural knowledge due to colonial enforcement and influence. Aboriginal Peoples continue to face a long and arduous journey to find and repair the remnants of their history, cultural knowledge, and practises. This disturbance of intergenerational systems of knowledge has had a profound impact on the social, emotional, and physical wellbeing of Aboriginal Peoples (3), thereby contributing to the ongoing experience of intergenerational trauma (4).

As Western development continues to expand and the demand for extractive natural resource industries increases, the disconnection between Country and community grows. Furthermore, extractive practises have been proven to contribute to the exacerbation of climate change effects, thereby inducing the prevalence of weather-induced risks such as extreme fire disasters (5). Studies suggest that climate change-induced weather events and natural disasters have increased the presentation of mental health diseases, such as sleep disorders, stress, anxiety, and depression, and may cause the development of more serious issues such as post-traumatic stress disorder and suicidal ideation (6, 7). According to the United Nations (8), Aboriginal Peoples, in particular, are among the first to face the direct consequences of climate change due to this close relationship with the environment and its resources. The cultural traditions and spirituality of Aboriginal Peoples are strongly connected to maintaining the wellbeing of Country (9), and the decimation of the land brings a profound sense of grief and loss as land is seen as central by many (10). However, this observation is rarely considered, and Indigenous voices are seldom included in national discussions on climate change. During the Native Title Conference of 2008 (11), Warwick Baird stated:

Climate change was impacting and was going to impact even more on Indigenous Peoples globally in a unique way because of this deep engagement they have with the land… In Australia, climate change policy is developing rapidly and Indigenous People are not included or their engagement is on a piecemeal basis (11).

In 2019/20, the frequency, intensity, and impact of natural disasters due to climate change became all too apparent when the catastrophic disaster dubbed the *Black Summer Bushfires* spread across Australia. This disaster was unexpected and nothing like Australia had ever experienced before (12). Occurring at the end of a severe drought, the bushfires resulted in unprecedented devastation across much of the Country, with estimates suggesting 14.5 million acres of land were affected (13). Bushfires, like other natural disasters, have long-term effects on the mental health of affected individuals and communities (14), and as previously stated, have a profound impact on Aboriginal Peoples. As such, a growing body of research into the psychological impacts of climate change began to evolve, and new developments of language emerged in response to the emotional experiences described by those affected. The use of pre-existing and recently coined terminologies emerging from this space, such as eco-anxiety (15), eco-angst (16), and solastalgia (17), began to appear in the respondent literature. A thorough examination of the development and definitions relating to these emerging terminologies revealed a particularly interesting correlation between the origins of solastalgia and the potential application of this term regarding Aboriginal-specific emotional responses. Subsequently, the term “solastalgia” was chosen as the basis of this review.

In alignment with the aim of this review to apply a decolonised, Indigenist lens, conventional scoping review processes have been adapted or replaced by generally unfamiliar Indigenist methodologies. Additionally, the conventional structure of this review has been modified to read as an *iterative* or *circular* storey.

## 2 Introduction

The term solastalgia was coined by the environmental philosopher Professor Glenn Albrecht (18). Solastalgia refers to the lived experience of distress caused by the loss of value and the desolation of one's home environment. This conceptual condition manifests in feelings of placelessness and being undermined by forces that destroy the potential for solace in these situations. Furthermore, solastalgia is defined as a form of homesickness experienced when one is still at home yet is affected by the destruction of one's home environment (19). The term solastalgia appears under the umbrella of “Psychoterratic States” (20), which is a concept defined as “the health relationship between the psyche and the biophysical environment” (20). During the term's conception, Albrecht explored the philosophical relationality of psychological states and environmental destruction after he identified the correlation between the prevalence of open-cut mining and drought in eastern Australia and the psychological impact on the surrounding communities (21). This relationality is reflected in the results of preliminary searches of current publications. Additionally, the term solastalgia was found to be discussed primarily in relation to the population as a whole rather than in the context of specific populations. Evidence of this finding was reflected in the resulting scoping reviews identified in the preliminary searches. The three scoping reviews identified presented little discussion of Aboriginal-specific content, thus contributing to the hypothesis that solastalgia has not often been discussed in the context of an Aboriginal perspective.

There was, however, one scoping review that showed potential. It was conducted with the aim of mapping the existing literature on solastalgia within an Australian context with a particular focus on Aboriginal and Torres Strait Islander experiences (22). Consequently, one of the key outcomes of the review was the recommendation that solastalgia should be further explored in the context of Aboriginal and Torres Strait Islander perspectives as a result of identifying the possibility of Aboriginal Peoples being particularly at risk of experiencing solastalgia. Additionally, the review recommends further exploration of the suitability of applying the term solastalgia to Aboriginal and Torres Strait Islander Peoples' emotional responses/experiences to environmental change (22). Furthermore, the review provided support for this current review by stating that future research into solastalgia would benefit from being conducted using a decolonised lens (22).

As corroborated by the results provided by Breth-Petersen et al. (22), an extensive search of the Pubmed, ProQuest, INFORMIT Indigenous, EBSCO, and Google Scholar databases determined that there was a significant gap in the literature that failed to include Aboriginal-specific discussion relating to solastalgia. Additionally, those in existence were primarily authored by non-Indigenous academics and were not reflective of a decolonised lens.

Given the importance of “place” and Country in Aboriginal cultures, the suggestion of further research into an Aboriginal perspective of solastalgia, and the recommendation of a decolonised review, we proposed that place-based lived experience of solastalgia in Aboriginal Communities should be a key aspect identified within the chosen publications of this review. Therefore, this study aims to provide a comprehensive scoping literature review of the concept of solastalgia from the perspective of Aboriginal Peoples' experience of the phenomenon whilst embedding a decolonised, Indigenist approach. Additionally, this literature review was conducted during the initial stages of the lead authors' (known hereafter as KLU) PhD project as a way of examining the scope of current solastalgia-related literature in the context of applying the concept to Aboriginal Peoples' experiences of negative environmental change. Before conducting this review, KLU developed an Indigenist understanding of the term by performing a modified heuristic inquiry study (23) (see Data Sheet 1).

## 3 Review questions

The objective of this review is to contribute to the narrative of decolonising discussions within the realms of Aboriginal experiences of climate change and explore the potential application of the term solastalgia to the experiences of Aboriginal Peoples. Research on decolonising practises suggests that co-researching methodologies should be included in designing and conducting research. This practise aspires to re-cover, re-cognise, re-create, re-present, and “re-search back” using Indigenous ontological and epistemological constructs (21). Therefore, with respect to embracing co-design, also known as Aboriginal participatory action research (APAR) (24), a discussion was held with the University of New England's (UNE) Aboriginal Advisory Committee, which consists of local Gamilaraay community members, facilitated by the stakeholders of the UNE Medical Research Futures Fund (MRFF) bushfire impact study. The community consultation resulted in a unanimous agreement that the preceding literature review should be conducted in a decolonised manner, applying an Indigenist lens, and include publications that focus on an Aboriginal connexion to the Country.

The questions that guide this review were collaboratively developed by the UNE Aboriginal Advisory Committee, authors, and academic associates of the research team who identify as Aboriginal or have extensive professional experience in Aboriginal mental health research.

The aim of this review is to:

- Apply a decolonised, Indigenist lens in the identification of publications, analysis methodologies, and resulting discussions;
- Explore the value of Aboriginal authorship, collaboration, and community participatory research methods in Aboriginal-specific climate change discussions;
- Map/outline how the concept of solastalgia as experienced by Aboriginal Peoples is revealed in the literature; and
- Examine the relationality of connexion to Country or place and solastalgia.

The following research questions guide the review:

1. How do Aboriginal peoples experience solastalgia?
2. Are there similar concepts of solastalgia discussed within Aboriginal-specific literature relating to the impact of environmental change?
3. What can we learn from the literature in terms of the distress associated with environmental change?

## 4 Eligibility criteria

### 4.1 Concept

The typical process of conducting a Higher Degree of Research (HDR) project requires the candidate to explore their chosen topic by conducting a literature review. The aim of the literature review process is to “map rapidly the key concepts underpinning a research area and the main sources and types of evidence available” (25). However, as evidenced by the initial searches conducted at the beginning of this literature review, published literature discussing solastalgia from an Indigenist perspective is few and far between. Therefore, the researchers conducting this literature review conceded that a conventional Western review process would be inappropriate. They were particularly pertaining to applying a decolonised, Indigenist lens.

Further examination of Indigenist methodologies suggests that autonomous perspectives derived from lived experience, cultural backgrounds, and prior cultural teachings are essential elements of postcolonial scholarship. As defined by Tsinnajinnie et al. (26), Indigenous research is “An intentional decolonisation process that we engage in as Indigenous individuals with many shared values linking our spaces together, inserting Native epistemologies, and honouring the reclamation of how and why we seek knowledge” (26). Consequently, unlike the usual process of conducting a literature review, the Indigenous researchers on this project convened in a series of virtual “yarning circles” to discuss the concept of solastalgia from a lived experience, Indigenist perspective and determining the best course of action for this review. These discussions were led by KLU, who had spent a period of time delving into the conceptual elements of solastalgia using heuristic inquiry, thereby beginning the scoping review process with a pre-conceived notion of how the term solastalgia may or may not reflect the deep sense of homesickness and loss related to environmental change as experienced by Aboriginal Peoples. Additionally, as posed by the team and later confirmed by Professor Albrecht himself, in an unpublished conversation with KLU in 2023, solastalgia is defined as an emotional response related specifically to the degradation of the biophysical environment. Biophysical means “involving biological or physical factors or considerations” (27), thereby limiting the potential of extending the term solastalgia to broader causes such as psychosocial factors that are inherently interconnected, for instance, the spiritual dimensions of an Aboriginal Persons relationship to Country.

Therefore, due to the possibility of alternative perspectives of the same concept, resulting in the potential of differing language, this review developed into a systematic process of inquiry designed to explore the basic underlying concept of the term.

### 4.2 Context

The context of this review is to examine the current existing published literature to form the basis of a Ph.D. project that will explore the experience of natural disasters, such as bushfires, by Aboriginal peoples. By developing an understanding of the possible emotional responses related to detrimental environmental changes. In particular, the application of the term solastalgia in consecutive publications.

### 4.3 Types of sources

As stated previously, the answers we seek are not embedded in Western philosophy or methodologies alone. They require an atypical, decolonised, Indigenist approach to draw out the information.

Throughout the numerous “yarning circles” held, the authors devised a list of appropriate inclusion and exclusion criteria to guide the literature selection process (see Table 1). Additionally, the authors considered both qualitative and quantitative studies, perspectives papers, and both peer-reviewed and grey literature. However, grey literature would only be included within this scoping review if it was determined to be from a reputable source.

**Table 1:** Inclusion and exclusion criteria for literature review screening.

**Inclusion**	**Exclusion**
• Peer-reviewed and/or grey literature, provided it demonstrates discussions that align with the concepts asked within the key questions; • Literature featuring discussions of the relationship between Country and a sense of loss as a result of environmental changes; • Aboriginal-specific literature, with discussions of Aboriginal perspectives or experiences; • Literature that includes discussions about the importance of Connexion to Country as an imperative element of Aboriginal cultural ties, kinship, and place-based positioning.	• Literature without a particular focus on discussions involving the experiences and perspectives of Aboriginal peoples; • Literature derived outside of an Australian experience; • Secondary literature; • Non-human related research; • Grey literature is found to have had no level of the peer-review process.

## 5 Methodologies and framework

No specific framework was initially developed to carry out the process of this review. Instead, this review retained a fluid and “living” working practise, developing autonomously with guidance from both Western and Indigenist methodologies. See Data Sheet 1 for an extensive Methodologies and Frameworks list and summary.

The resulting process was conducted as follows:

**A decolonised, Indigenist Scoping Review Process**

1. KLU dedicated 6 months to thoroughly understand the concept of solastalgia by conducting a period of modified, creative, Indigenist heuristic inquiry (23). Throughout this, numerous conversations were held with esteemed elders, Gamilaraay community members, climate change impact experts, and the researchers involved in conducting the scoping review. During this time, three paintings were created as a way of conceptualising, visualising, and translating gained knowledge;
2. The researchers conceded that in addition to the guidance of “Quandamooka” by Martin and Mirraboopa (28), the Joanna Briggs Institute methodology for scoping reviews (29) would be consulted in the literature review design. This guideline informed the review process and the structure of the final report. Additionally, the PRISMA-ScR reporting guidelines (30) were chosen to create a modified display of the search results;
3. An *a priori* (29) protocol (unpublished) was developed as a “living” document, thereby changing as the direction, content, and process change;
4. Initial database searches were conducted with the following search terms:
5. “(solastalgia OR solistalgia) AND (indig^*^ OR aborig^*^) AND (kinship OR connexion OR placelessness OR climate change) AND “Australia.”
6. This search was performed with the assistance of the UNE librarian and produced inadequate results;
7. The heuristic inquiry process led KLU to deduce that publications relating to similar concepts defined by the term solastalgia may be present; however, the language used may vary due to the potential deviation from contemporary Western nuances. To further explore the concept of solastalgia and determine the appropriate means of conducting this review, the researchers convened with the UNE Aboriginal Advisory Committee in an informal “yarn.” Committee members were provided with an in-depth explanation of the term solastalgia and were asked to “*yarn-up*” (discuss) their understanding of the word whilst allowing space for questions and debate. It was determined that the Aboriginal philosophy of connexion to Country may result in a greater sense of loss associated with environmental change. Additionally, the elders suggested that the experience of environmental change may indeed impact Aboriginal peoples emotionally, and also physically and spiritually, and result in widespread changes in community dynamics, alongside a disconnection from the Country. Additionally, this meeting assisted in determining the appropriate search terms for the second search and the inclusion/exclusion criteria for the chosen literature;
8. Individual “yarns” were had between KLU and the Aboriginal members of the research team, thus determining the methodologies that would inform the review process, confirming the appropriate research questions, and validating the search terms;
9. Researchers KLU, RS, and JD met virtually to conduct the database search. Before beginning the search, the researchers discussed, in detail, the pre-determined concepts within solastalgia and the inclusion/exclusion criteria. JD performed the searches whilst screen sharing with the other researchers. The results were extracted and loaded into EndNote (31) software. Duplicates were removed, deductions were made via title search and year, deductions via abstract content, and finally, full-text skimming produced a total of 35 publications (see Section 6; search strategy);
10. Each of the researchers read the publications independently. A secondary virtual meeting was held in which each of the researchers produced their chosen literature. A lengthy discussion took place about the appropriate direction for the review and accompanying texts. Finally, six of the publications were chosen for the scoping review;
11. KLU uploaded each of the six publications into NVIVO (3) software and used the software function of determining the “most common words” (see Data Sheet 2). Additionally, a data extraction table (see Data Sheet 3) was collated, thereby informing the process of determining the recurring themes within the texts, which were further examined and correlated using a PAGER Framework (32) table (see Table 2);
12. Researchers KLU, RS, and JD conducted a final virtual meeting to discuss the extracted data and resulting identified themes. Each of the researchers was in agreeance with the themes, and the results were forwarded to the project team;
13. KLU consulted with two cultural mentors during this time to verify the direction of the review. KLU corresponded with the research team via email with updated versions of the report for validation, feedback, and input. KLU continued to perform heuristic inquiry, discuss solastalgia widely, and revisit the search terms for updated publications. Subsequently, a publication by McNamara and Westoby (33) was discovered, and despite the inclusion criteria stating that texts should reflect Aboriginal perspectives, the text was deemed to be relevant as it discussed Torres Strait Islander experiences of solastalgia. Thereby, the content was analysed and subsequently added to the review. Additionally, the literature review by Breth-Petersen et al. (22) was identified and included in this final report as validation for conducting a decolonised, Indigenist scoping review process.

**Table 2:** PAGER framework table.

**Pattern**	**Advances**	**Gaps**	**Evidence**	**Research recommendations**
Authorship of Country and traditional knowledge	- Authorship of Country - Collaborative research methodologies such as Aboriginal Participatory Action Research (APAR). - Increasing frequencies of Aboriginal and Torres Strait Islander researchers, contributing to Aboriginal-specific research. - Where research involving Indigenous fields of study are conducted by non-Indigenous researchers, co-design methods or consulting is used.	- Database search identified four texts, currently published, featuring Country as lead author. - Traditional knowledge's infrequently cited by the following selected publications, “Strange Changes,” “We're the same as the Inuit,” “Future sea changes,” and “Solatalgia and the Gendered Nature.”	- Current published works featuring Country as the origin of knowledge.	- Prime examples of Country authorship will encourage the development of future literature to adopt a similar approach. - It is recommended that a continued effort is required to involve Aboriginal researchers in Aboriginal-specific research. - Utilising Indigenist research methodologies. - APAR methods of co-design.
Storytelling as methodology	- The adoption of storytelling methodologies present throughout most of the selected publications. - Storey utilised as a translation methodology	- The publications that featured “western” methods of data collection, selected relevant, but fractured samples of text, rather than featuring the storey in it's entirely. This, potentially misrepresenting the context.	- Each of the publications featuring Bawaka Country as lead author, succeeded in interpreting Traditional systems of knowledge in a comprehensive yet accessible manner	- Indigenous Data Sovereignty recommends that storeys should be featured in their entirety
Terminology	- Proven theory of similar terminologies pertaining to climate change are present in all chosen texts, whilst demonstrating that some terminologies may differ, depending on the context or Country of origin.	- Database searches using direct terminologies used in climate research context did not produce extensive results.	- By broadening the search terms to suit Indigenist research, a wider body of work was discovered.	- Future Aboriginal-specific climate change research, should feature broad terminologies, as well as climate-specific terms. Thus, allowing them to be more easily discoverable.
Aboriginal perspectives and experience of environmental change	- The selected publications that discuss environmental change, specifically, features elements of Aboriginal perspectives from direct sources.	- “Aboriginal” perspectives are a generalised term, and does not allow for alternative perspectives derived from factors such as, Country of origin.	- Four texts depict environmental change as distressing. - The remaining texts conceptualise climate change alternatively.	- Further research should explore the alternative perspectives, Cultural knowledge's, philosophies and ontologies of a broad range of Aboriginal peoples/communities.
Connexion to Country and environmental changes	- The relationship of connexion to Country, discussed extensively throughout the texts. - The impact of environmental change effecting this relationship is explored throughout the texts.	- There discussions feature within the texts, that do not explore the connexion between “grief and loss,” pertaining to a connexion to Country.	- Each of the texts feature discussions of environmental change being observed by Aboriginal peoples.	- It is recommended that future research of Climate Change that discusses the impact on Aboriginal peoples, should feature the impact of relationships to Country.
Adapting to environmental change	- Depictions of resilient attitudes towards the future, were evident throughout each of the texts. - Climate change adaption discussions and planning were evident throughout most of the texts.	- The literature rarely identified currently implemented and successful adaption practises.	- Most of the texts suggest immediate adaption conversations between community and key change makers i.e., policy writers	- Further research is recommended to explore potential adaption strategies.

## 6 Search strategy

A strategic scoping review protocol had been initially developed as a fluid, living document. Therefore, the process of conducting this review was informed by the discoveries, epiphanies, and insights gained at the time of its development.

As per the recommended scoping review guidelines outlined within the Joanna Briggs Institute methodology for scoping reviews (29), an initial search of the literature was conducted with the assistance of the UNE librarian. The initial search terms (see Section 5; methodologies and frameworks) produced minimal appropriate results. However, this was to be expected. As substantiated by Martin and Mirraboopa (28), the initial search would inevitably produce “sources produced by people and by other entities in the context of Country.” Additionally, Martin and Mirraboopa (28) recommend that “Primary and secondary sources by non-Aboriginal people should also be reviewed, keeping in mind the cultural assumptions, standpoints and biases of the author.” Furthermore, the use of the term “solastalgia” in the literature pertaining to an Aboriginal experience is uncommon, as confirmed by the subsequently identified literature review by Breth-Petersen et al. (22).

Consultations with Aboriginal community members and the research team resulted in the selected search terms outlined in Section 5 (methodologies and frameworks).

These terms were deemed to be culturally appropriate and reflected the notion of connexion to Country as an important factor of understanding the emotional responses of Aboriginal Peoples as derived from environmental degradation.

As stated in Section 5 (methodologies and framework), a revised search was conducted by KLU, RS, and JD, using the new search terms in the following databases: PubMed, ProQuest, INFORMIT Indigenous, and Google Scholar. The initial results identified 688 citations, after which a screening process was conducted. A title search deducted a portion of the results, followed by a reduction process that kept texts adhering to the following criteria: Date ranged between 2005 and 2022, geographical location restricted to Australia, peer-reviewed texts, and language limited to English. This reduced the search results to 38 citations identified from the databases listed and a further 109 citations identified from Google Scholar. Additionally, three texts were identified through citation searches. Finally, a screening process was conducted whereby duplicates were removed, and the remaining citations were further screened for relevancy, reducing the total to 129.

Each of the remaining citations and abstracts was screened to determine the relevance to the inclusion criteria (see Table 1). A total of 35 texts remained for full-text screening, conducted independently by KLU, RS, and JD. It should be noted that two of the academics involved in the screening process identify as Gamilaraay.

**Figure 1:**
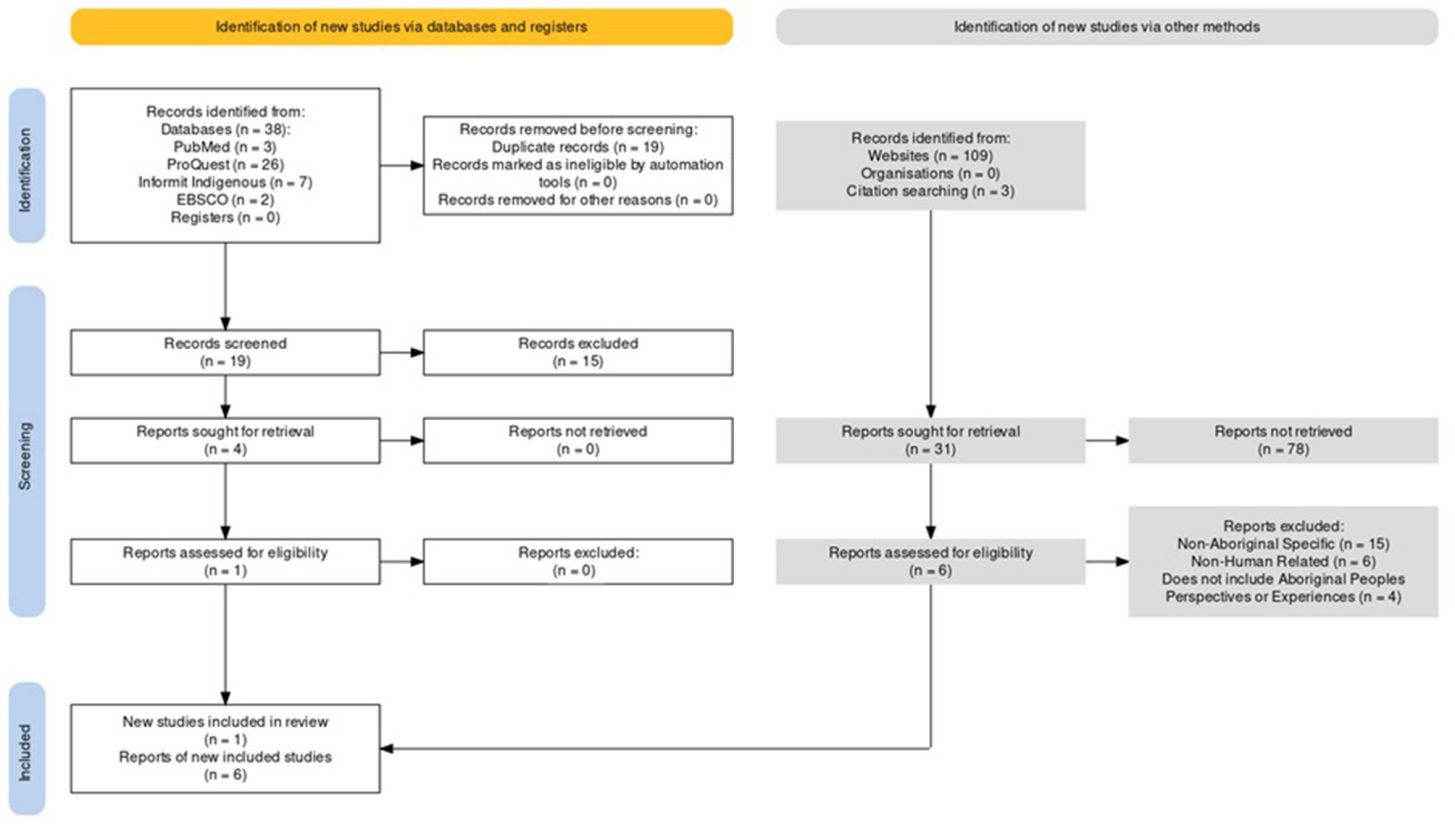
PRISMA flow diagram.

A subsequent virtual meeting was held, whereby KLU, RS, and JD presented their chosen texts and outlined their reasoning. A discussion concluded that the six remaining texts adhered to the inclusion criteria. Additionally, the texts chosen discussed topics and were structured in a way that fulfilled our prior queries, specifically discussions of connexion to Country, Aboriginal perspectives, climate change and/or environmental changes, Aboriginal philosophy, and instances of co-creation. The search process and results are presented using the following PRISMA Flow Diagram (30) model (Figure 1):

During the synthesis of the review, KLU continued to revisit the database search results to identify potentially relevant publications. Consequently, the literature review by Breth-Petersen et al. (22) was identified during a subsequent search, which was determined to be a valuable contribution to the subject matter. However, due to the nature of the inclusion criteria, this publication was excluded from the review, although relevant information within this publication was considered. Additionally, a second publication was identified post-analysis, which primarily featured Torres Strait Islander experiences and perspectives. Regardless of the inclusion criteria, due to the relevancy of the content aligning with the research questions, this publication was included for review. In conclusion, the following seven publications were identified for discussion:

- Petheram et al. (34), “Strange changes:” Indigenous perspectives of climate change and adaptation in NE Arnhem Land (Australia);
- Bawaka Country et al. (35), Gathering of the Clouds: Attending to Indigenous understandings of time and climate through songspirals;
- Nash et al. (36), We're the same as the Inuit!: Exploring Australian Aboriginal perceptions of climate change in a multidisciplinary mixed methods study;
- Petheram et al. (37), Future sea changes: Indigenous women's preferences for adaptation to climate change on South Goulburn Island, Northern Territory (Australia);
- Bawaka et al. (38), Co-becoming Bawaka: Towards a relational understanding of place/space;
- Bawaka et al. (39), Caring as Country: Towards an ontology of co-becoming in natural resource management; and
- McNamara and Westoby (33), Solastalgia and the Gendered Nature of Climate Change: An Example from Erub Island, Torres Strait.

Seeing as though five of the seven texts feature recurring lead author identifiers, throughout the discussion of this review, the publications will be referred to by the key features of the title, i.e., Petheram et al. (37) will be referred to as “Future sea changes” to simplify readability.

## 7 Data extraction and analysis

As stated in Section 5 (methodologies and framework), KLU used EndNote (31) software to determine the “most common words” within the combined texts (see Data Sheet 2). The most notable and frequent words with their accompanied derivatives were:

- Changing etc.;
- Climatic etc.;
- Humans etc.;
- Community etc.;
- Country etc.;
- Becoming etc.;
- Indigenous;
- Adaptive etc.;
- Relations etc.;
- Time etc.; and
- Understandings.

By identifying the most common words, themes begin to emerge. Additionally, each of the seven texts was thoroughly examined, the analysis of which informed the development of a glossary of key terms (see Table 3). Key terms were selected by reviewing the “most common words” table (Data Sheet 2), combined with the results of the database search terms and a reflection of previous discussions. Finally, full-text screening resulted in the research team identifying “topics of interest.” Key publication information and the “topics of interest” headings were incorporated into a data extraction table (Data Sheet 3), as listed here:

- Author, year of publication, and Country;
- Aim/purpose;
- Methodology/methods;
- Key findings;
- Connexion to Country;
- Knowledge translation/traditional knowledge and practises;
- Climate change; and
- Adaption.

**Table 3:** Glossary and context of key terms.

**Term**	**General context**
Country	Often used to describe the land and all that it encompasses, usually in reference to ancestral lands. Also known as “land” and “place.”
Connexion to Country	The spiritual, physical, cultural, social connexion to “Country.” This encompasses language, Lore, Kinship and Ceremony.
Adaption	The process of adapting to the changing environment.
Land	The ecosystem that sustainably supports a person or community.
Place	Also known as “home” and “Country.” Often used to describe a specific area and the connexions associated.
Placelessness	Often used to describe a sense of disconnection to Country, community and culture.
Kinship	The complex relationship with family, community and Country.
Co-becoming	The interconnected relationship to all things.
Loss and grief	The painful feelings associated with spiritual, physical, social and emotional disconnection from community, Culture and Country.

The final column was reserved for recording emerging themes or patterns, as identified through performing a virtual “yarn-up” by the research team. Additionally, whilst discussing the findings of the data extraction table and revisiting the original research questions for this review, the research team compiled the emerging themes into a PAGER framework (32) table. This framework is a method of identifying the gaps between the key findings of the data analysis and the possibility of answering the research questions. The PAGER framework (32) matrix is typically used as a method of charting “patterns” or identified themes, “advances,” i.e., significant discoveries, “gaps” identified within the literature, “evidence,” i.e., for practise, and “research” recommendations. The identified themes were compiled into the “pattern” column of the table, and the proceeding information was extracted from an additional revision of the included publications, followed by an informal meeting of the research team, whereby the researchers were in agreement with the resulting information recorded into the following table.

The extracted information presented within the PAGER table informed the subsequent discussion. Furthermore, the table provided valuable insights that were included in the final recommendations of this review.

## 8 Discussion

### 8.1 Authorship of Country and inclusion of traditional knowledge

#### 8.1.1 Significance to the review

The selection criteria specifically state that eligible articles will feature Aboriginal perspectives or experiences, with discussions of connexion to the Country as an imperative element. The act of attributing Country with authorship, demonstrated reliability of the information included within the paper by evidencing the original source.

#### 8.1.2 Evidence

To align with the value of cultural responsiveness in Aboriginal research, where possible, researchers should adopt co-design methodologies. The inclusion of Aboriginal peoples as co-researchers encourages Aboriginal Peoples to be empowered and self-determinant and allows communities to control their data, i.e., storeys, cultural knowledge, and art (24). Additionally, colonial influences perpetuated by Western academia can result in disinformation and misrepresentation of ideologies and lead to harmful deficit discourse narratives. Dudgeon et al. (24) illustrated that “The grand narratives of colonial nation states have served as mechanisms to perpetuate a false narrative about a vast and unclaimed territory ‘terra nullius' which has fuelled and sustained an epistemic erasure of the trauma, destruction and oppression being imposed on and experienced by Indigenous peoples globally and in Australia.” Therefore, by adopting co-design practises, interpretation of results and research dissemination will have greater accuracy and reflect a better understanding of the topic. In recent times, researchers have begun adapting Western research methods to be more inclusive, culturally sensitive, and responsible. As conceded by the article “Strange changes” (34), “The importance of respect for Indigenous views and their incorporation in development planning was emphasised by research participants.” Similarly, “We're the same as the Inuit” (29) states that “Indigenous community involvement was integral from the planning stages through to the data collection, analysis and dissemination of results.” This practise is essential to improving current Aboriginal-specific research standards, and due to this importance, this factor was included in the considerations of the publications identified for review.

An excellent example of a collaborative design that led to informative and accurate reflections on Aboriginal ways of “knowing, being, and doing” (21), is the paper titled “Co-becoming Bawaka: Towards a relational understanding of place/space” (38). Not only does the authorship acknowledge Country as the lead author and creator of the knowledge origins, but the authors describe the inherent relationship. Furthermore, the study describes the non-Indigenous research partners as being “adopted” into the family of the traditional owners of Gumatj (including Bawaka) Country (38), thereby permitting the non-Indigenous authors to learn from Country and discuss shared knowledge through the teachings passed on by the Gumatj elders. The non-Indigenous authors stated that “Bawaka enabled our learning, our meeting, the storeys that guide us, the connexions we discuss and has, indeed, brought us into being, as we are, and continue to co-become, today” (38). Similarly, reflected in the sentiments of “Caring as Country” (39):

Acknowledging the authorship of Bawaka Country is important as it decentres the privileging of human authors as the only beings able to control and create, as the sole deciders of content and structure, and opens up opportunities for reimaging and co-creating not only how we write about NRM (natural resource management) but how we think about and practise it (39).

Acknowledging the Country as the origin of traditional knowledge is a responsibility of both Aboriginal Peoples and those who are gifted the knowledge. This element is important to acknowledge and respect the continued practise of caring for Country and acknowledging ontologies that believe in a co-becoming with Country. Additionally, the act of acknowledging and recognising the origins of knowledge demonstrated respect for the non-linear, spoken, and unspoken connexion of all to the Country. Furthermore, accurate representation and ethical use of Indigenous knowledge's pertain to valuing the rights of Indigenous Peoples. The right to determine the use and publication of Indigenous knowledge is recognised as “Indigenous Data Sovereignty” (40), described by Kukutai and Taylor (40), as “the inherent and inalienable rights and interests of Indigenous Peoples relating to the collection, ownership and application of data about their people, lifeways and territories.”

For example, the study “Future sea changes” (37) concludes that “Findings of this research suggest that in engaging with Indigenous communities on planning for adaptation, it will be essential to discuss climate change and adaptation in ways that acknowledge differences in knowledge systems” (37), thereby reiterating the value of Indigenous knowledge and actively respecting the rights of Aboriginal participants. By valuing Indigenous data sovereignty, researchers actively partake in exercising decolonising practises.

### 8.2 Storytelling as methodology

#### 8.2.1 Significance to the review

Storey is a standard facet of data collected within Indigenous research studies. Similarly, storey is a commonly used Indigenous research methodology. The inclusion of storey substantiates the Aboriginal participation within the study and/or paper.

#### 8.2.2 Evidence

*Storey* was identified as one of the recurrent themes throughout the seven texts. “Caring as Country,” (39) “Co-becoming Bawaka,” (38) and “Gathering of the clouds,” (35) each use *storey* published in their entirety as a method of conveying Yolηu (Aboriginal peoples of northeast Arnhem Land) ontological philosophies and knowledge. Similarly, “We're the same as the Inuit,” (29) “Future sea changes,” (37) “Strange Changes,” (34) and “Solastlagia and the Gendered Nature” (33), feature storey; however, they include fractured interview excerpts rather than whole interview transcriptions, which consequently may lead to the misrepresentation of information and context. Kovach (41) states that “storeys hold within them knowledge's, while simultaneously signifying relationships.” Martin and Marriboopa states (28) that “Storeys are vessels for passing along teachings, medicines, and practises that can assist members of the collective.” Therefore, selective information could misinterpret or devalue the information, and interconnexions of the storey may be missed. Interestingly, it calls to question the accuracy of the information represented within a literature review, given that the undertaking of a review is influenced by a systematic bias.

As previously stated, respecting Indigenous Data Sovereignty means valuing the rights of traditional knowledge; therefore, Indigenist research methodologies, such as APAR, allow Aboriginal Peoples to control the use and dissemination of their “data.” The practise of extracting key features from a storey is rarely supported by Indigenous researchers as “storeys can never be decontextualised from the teller” (28). Furthermore, Aboriginal storey is irrevocably Indigenous knowledge as derived from the Country. By positioning the storey within its Country of origin, one respectfully acknowledges the unique characteristics, culture, and traditions of that Country. For example, “Gathering of the clouds” (35) tells a storey of the *Wukun* (gathering of the clouds) songspiral, which is unique to Bawaka Country. “Solastalgia and the Gendered Nature” (33), acknowledges that the storeys included in their studies originate from Erub Island of the Torres Strait.

The way in which the storey is documented or translated may lead to differing conclusions. The storey of *Wukun* (35) comes directly from the Country/community and describes a traditional knowledge system. *Wukun* (35) anticipates the ebbs and flows of weather patterns by situating oneself within that system, as part of that system. Acknowledging that in Yolηu ontology, distressing environmental circumstances will eventually be satiated, “And through the tears of the rain, grief may be stilled” (35). Whereas a mixed method, “Western” structured study, such as “We're the same as the Inuit” (29), identifies unabated distress over environmental change, stating that “It would also appear that deep connexion to and identification with the ‘natural' environment understandably increases not only concern and distress with respect to impending and dramatic climate change, but also collective self-efficacy and resolve” (29). This may be an example of storey translated directly from Country, differing from systematic, structured processes, resulting in conflicting sentiments. Otherwise, it may just be an example of alternate perspectives of environmental change. For example, the term “Indigenous” is a common term used to describe the Indigenous Peoples of Australia; however, depending on the preference and perspectives of the referred parties or context of the terms used, “First Nations Peoples” may be preferred. However, it is widely accepted that when appropriate, the specific nation of the referred party should be alternatively used, thus demonstrating the inability to group the preferences and perspectives of *all* Aboriginal Peoples and highlighting the need to explicitly detail the origins of the illustrated perspectives. In acknowledging this fact, we then pose the question, is there truly a way to determine the appropriate use of the term solastalgia as an emotional response experienced by Aboriginal Peoples in a generalised context?

### 8.3 Terminology

#### 8.3.1 Significance to the review

It was hypothesised that a wealth of literature pertaining to the grief and loss associated with environmental change might be difficult to locate due to the use of alternative language. Therefore, a thorough examination of the language present within the chosen articles was warranted.

#### 8.3.2 Evidence

It was hypothesised that change discussions might typically be disseminated in “English,” which may impact translatability, therefore limiting the number of results in the database searches of this review based on this factor. Consequently, an analysis of significant “most common words” was conducted, as contextualised and understood throughout each of the seven publications. The results of which are presented in the following glossary.

The general context of each key term, as described in this glossary, indicates that although the term solastalgia only appears in the text “Solastalgia and the Gendered Nature” (33), the correlating words and context of the terms that are often associated with the concept of solastalgia, are frequently exhibited in the remaining texts, thereby illustrating that language/terminology barriers may inhibit search results.

The definition of solastalgia, as previously discussed, is bounded in the context of the “biophysical” environment. This is a point of connexion among academics, mainstream media, and the general public, all of whom have differing interpretations of the term, thereby calling into question the context in which solastalgia is used.

One participant of the study “Solastalgia and the Gendered Nature” (33), which explores the experience of solastalgia, reflects upon their interpretative artworks by stating that she felt “great sadness as she sensed that her identity was changing” as a result of experiencing changes in her home environment. A factor that is associated with solastalgia, as evidenced by Albrecht (17), states that the experience of solastalgia may lead to “the erosion of the sense of belonging (identity) to a particular place” (17, 33). However, the notion of solastalgia being purely a response to the biophysical environment is explored by the researchers stating, “interviews explored not only the biophysical impacts of environmental and climate change but also potential emotional, Cultural and psycho-social impacts” (33); thus questioning the participant's sentiments as being a response to the biophysical environment, as it may in fact be evoked by factors such a deep connexion to Country. However, the study fails to include substantial details within this context, thereby highlighting the importance of including full transcripts to corroborate the context of the information. Additional details may have provided terminologies that would have given evidence to determine the source of the notion that their “identity was changing” (33).

In the study “Future Sea Changes” (37), participants involved were unfamiliar with the term “climate change.” Many of them stated that English was not their primary language. It was identified within the study that participants had a “limited understanding of western concepts and English language terms” (37), specifically “associated with climate change and why change was occurring” (37), demonstrating the importance of language and translatability. Similarly, the study “Strange changes” (34) noted that “Although all participants were familiar with the term climate change and had many ideas relating to its occurrence and potential changes, it became evident that many were unclear about western notions of the concept.” Furthermore, “Strange Changes” (34) stated that most participants “said they had heard of the term ‘climate change' through media, mostly television;” however, two participants appeared to distance the concept from their own personal experiences of changes within their home environment by associating their understanding of climate change with “Polar caps melting, affecting polar bears” (34). This highlights that societal and personal factors may influence the ability to translate Western terminologies into familiar language and contexts.

Alternatively, the publication “Co-becoming Bawaka” (38) offers a different scenario. Throughout the study, the term “climate change” is frequently used, and its context is understood. “Co-becoming Bawaka” (38) repeatedly relates “climate change” to Yolηu ontologies. As evidenced by the statement:

While climate change may appear as a quintessential “unbounded” phenomenon, gurrutu (a complex Yolηu kinship system immersed in the practise of digging ganguri or yams) will tell us that it is only ever manifested in grounded ways, such as cyclones, storm surges, government reports and protest marches, that are themselves linked to enduring place-based patterns of kinship and responsibility (38).

Consequently, it confirms that Yolηu Peoples are familiar with the concept of climate change by describing the relationality of the term to complex cultural ontological philosophies and knowledge systems, thereby reiterating the possibility that the participants of the “Strange changes” (34) study may in fact be familiar with the phenomenon of climate change. However, their interpretations of the concept may be embedded in traditional language and cultural knowledge rather than English.

### 8.4 Aboriginal perspectives or experience of environmental change

#### 8.4.1 Significance to the review

The inclusion criteria states that eligible studies would include Aboriginal perspectives or experiences. Whilst discussions featuring the relationship between Country and a sense of loss as a result of environmental change would be preferable, this review considered a general discussion of environmental change as appropriate.

#### 8.4.2 Evidence

As previously discussed, co-research methodologies such as APAR (24) are an essential component of determining Aboriginal perspectives. If we are to begin practising research that positions the Country as the origin of Traditional knowledge, the following discussions of “Aboriginal perspectives or experience of environmental change” will reflect this sentiment as an example of “best practise.”

Storeys derived from Bawaka Country, as told by “Caring as Country” (39), “Co-becoming Bawaka” (38), and “Gathering of the Clouds” (35), are centred around Yolηu ontological philosophies of time, place, and space. They are illustrating the differences between Yolηu concepts and comprehension abilities and highlighting the limitations imposed by “Western” constructs of conceptual understanding and self-positioning. These storeys are gifted to the reader as a way of educating the public about the alternative perceptions of our current space, particularly with regards to environmental change by guiding the reader to deconstruct their pre-conceived ideologies and ontologies. Interestingly, this particular sentiment may allow us to consider the possibility that the Yolηu practise of co-becoming Country, weather, and climate systems may reflect that in Yolηu ontology, acceptance of what was, what is, and what will be could argue the existence of solastalgia within Yolηu philosophy. As illustrated by “Co-becoming Bawaka” (38), “understanding place/space as co-becoming has the potential to inform discussion around global environmental change as it may ‘significantly' expand people's sense of what Earth futures are desirable and achievable.” Continued by, “climate change is not abstract—it too is part of a more-than-human co-becoming which is part of ‘us' (in the broadest more-than-human understanding of that word) and as such demands response and responsibility” (38).

Alternatively, “We're the same as the Inuit” (29), “Future sea changes” (37), “Strange changes” (34), and “Solastlagia and the Gendered Nature” (33) discuss the emotional responses towards environmental change, whilst featuring language that is concurrent with standard climate change terminology. Each publication does, however, reflect differing perspectives of the individual experiences of their participants towards environmental change, which are mostly reflective of a negative perception.

### 8.5 Connexion to country and environmental changes

#### 8.5.1 Significance to the review

The inclusion criteria state that connexion to Country is imperative. However, as acknowledged, individuals have unique perspectives and relationships with their environment. Therefore, this detail is generalised to include both Western and Indigenous terminologies and concepts.

#### 8.5.2 Evidence

It is important to note that the act of translating a storey derived from traditional languages into written English is a difficult process, as the Indigenous philosophies of “knowing, being, and doing” (23) that underpin the context of the storey are grounded in complexity, especially when discussing the complexity of Aboriginal philosophies of connecting to Country. Some narratives are exceedingly exceptional at expressing the inexpressible in such a way that allows the reader to comprehend the context and concepts behind the storey. For instance, “Co-becoming Bawaka” (38) interprets the act of digging for *ganguri* (yams) in a way that allows the reader to understand the complexity of Yolηu systems of knowledge, demonstrating a skill that is infrequently well-executed; a prime example of decolonised, Indigenist methodologies of interpreting and translating the storey. “Strange changes” (34) illustrates the exploration of the concept of “place,” using both qualitative and quantitative methodologies. Concluding that:

All research respondents exhibited strong connexion to place, and sensitivity to natural landscape. They talked of the social, cultural and physical features in their landscape in a multi-layered and interconnected way. Strong emphasis was placed on the importance of maintaining traditional knowledge, values embodied in nature, health of future generations, and the landscape (34).

Similarly, the study “Solastalgia and the Gendered Nature…” (34) considered this intrinsic relationship by affirming that “Country” denotes not only the physical elements of sea and land but also the spiritual and cosmic relationship that Torres Strait Islander Peoples have with the natural environment.

For the most part, the sentiment of connecting to the Country is positively affirmed throughout the seven texts. However, in some cases, this relationship is perceived as being heavily impacted by environmental degradation. For instance, “Strange changes” (34) states that:

Participants often talked of (a local) mine's negative impacts on the environment, and how this affected them to their core. The study recalls a participant singing a song they composed titled, White man's mine, which proclaims “Yolngu people have so long cherished the land in our culture we love to share, but you are taking it over again, what do we get in return?” (34)

Similarly, “Solastalgia and the Gendered Nature…” (33) claims that environmental changes appear to be “destabilising traditional ways of knowing and reading the landscape, including the predictability of weather, seasons, tides, and plant and animal cycles.”

Differing descriptions of personal relationships with Country as potentially being affected by negative environmental changes reaffirms the theory that the experience of what could be perceived as solastalgia is dependent on individual perspectives based on lived experience and personal philosophies.

### 8.6 Adapting to environmental change

#### 8.6.1 Significance to the review

Research question three asks, “What can we learn from the literature in terms of the distress associated with environmental change?” The theme of adaption to changes within the environment was present and therefore included within the discussion of this review.

#### 8.6.2 Evidence

An objective of the study “We're the same as the Inuit” (29) was to explore what the future holds for remote Aboriginal communities in terms of climate change adaption by developing an understanding of how they perceive the imposing threat of environmental change. There is evidence to suggest that without adequate research into appropriate adaption strategies, these communities will be disproportionately impacted by climate change. A result of this may be the potential of forced relocation from ancestral lands. “We're the same as the Inuit” (29) states that “despite the increasingly grim predictions of change for inland Australia, and the increasingly harsh conditions endured by its residents, many Aboriginal Peoples do not welcome the prospect of moving away from their communities and their countries as an adaptation strategy.” Whereas, “Solastalgia and the Gendered Nature…” (33) makes a different argument by stating that, “Torres Strait Islanders have adapted to biophysical changes in the environment for millennia,” followed by “Torres Strait Islander Peoples have co-evolved to adapt between the island, sea, nature and their culture.” “Solastalgia and the Gendered Nature…” (33) do however pointedly state that “the future of the islands is uncertain and the unpredictability the changing environment may impede attempts to stay connected to their ancestral lands.” Thereby, it is noted that even though Torres Strait Islander Peoples over many generations have adapted to environmental change, the current risks posed by climate change on their Islands may exceed previous adaption strategies, highlighting the imperative need for developing region-specific adaption strategies sooner rather than later.

Whilst climate change-associated risks are similarly intensifying across all regions, adaptation strategies are still feasible for remote Aboriginal communities, as opposed to those impacted by rising sea levels. “Strange changes” (34) expresses that the effects of climate change on Aboriginal Peoples causes a “loss of self-esteem, independence and self-sufficiency, loss of traditional knowledge and imposition of other cultures ways and values and loss of culture and dignity through lost contact with natural surroundings” (34). Consequently, participants were asked to create a list of strategies that may improve the adaptive capacity of their communities. One suggestion was that returning to traditional practises “would allow communities to be less dependent on services and maintain culture” (34). Concurrently, “We're the same as the Inuit” (29) states that “nature-connecting activities could be an important integral component in adaptation strategies to enable improved wellbeing for greater resilience to uncertainty under future climate change and food insecurity” (29). Whilst “Solastalgia and the Gendered Nature…” (33) reaffirms this position by stating, “whilst experiences linked to a changing climate can contribute to Solastalgia, communities that resist or adapt to such changes can maintain their sense of place and identity.”

## 9 Conclusion

The aim of this scoping review was to develop an understanding of how Aboriginal Peoples experience solastalgia by using a decolonised, Indigenist lens. When database searches were conducted, “Solastalgia and the Gendered Nature...” (33) was the only study that specifically referred to the term solastalgia. However, given the relatively new emergence of the term solastalgia, it is not surprising that there is little evidence that the term has been used in Aboriginal-specific literature derived from Australia.

“Solastalgia and the Gendered Nature…” (33) describes the experience of solastalgia as a sense of “homesickness,” evident in the depiction of declining familiarity with the Country by Aboriginal participants who continue to live on traditional lands. Alternatively, “Co-becoming Bawaka” (38) suggest that climate change is a “quintessential ‘unbounded' phenomenon where the Yolηu ontology of *gurrutu* (digging for yams) will tell us that it is only ever manifested in grounded ways,” and that they themselves are “linked to enduring place-based patterns of kinship and responsibility” (38). Therefore, calling into question the applicability of solastalgia as a generalised emotional response by Aboriginal Peoples and arguing that solastalgia is dependent on the individuals perspective.

In conclusion, the decolonised, Indigenist lens with which this scoping review was conducted has identified a correlation between the concept of solastalgia and a sense of disconnection from the Country caused by environmental changes. However, it determined that at this stage, there is inconclusive evidence that Aboriginal Peoples, in general, experience solastalgia. Solastalgia can, however, be perceived as an emotional response experienced by an individual based on their personal philosophy. Therefore, further research in this space is encouraged.

## 10 Recommendations

It is recommended that researchers continue to explore the impact of climate change-related disasters and environmental change as experienced by Aboriginal Peoples. It is recommended that future research should include co-design methodologies and/or Aboriginal authorship. By acknowledging the relationship/kinship of Country and community, the direct inclusion of Aboriginal Peoples would greatly benefit future research projects in this space. Furthermore, the deep inherent connexion between Aboriginal Peoples and Country reveals a distinct perspective of environmental change and the potential experience of solastalgia.

The discovery of literature authored by Country opens the door for the advancement of research led by traditional knowledge, therefore immersing research outputs with Aboriginal ontological philosophies. The contribution of cultural knowledge and practises in research provides an alternative position to the relationship between the environment and Aboriginal communities. Additionally, the exploration of Aboriginal systems of Country and climate is recommended as a valuable addition to future research on climate change and the increasingly frequent, subsequent natural disasters. It is recommended that future research into the impact of climate change as experienced by Aboriginal peoples explore the use of traditional healing practises in response to both the personal and communal effects of environmental change. Continued research in this space will aid the development of post-disaster planning and management whilst informing the development of future adaption policies. Finally, it is recommended that the inclusion of a decolonised, Indigenist lens is essential for future research in this space.

## Data availability statement

The original contributions presented in the study are included in the article/Supplementary material, further inquiries can be directed to the corresponding author.

## Author contributions

KUp: Conceptualization, Data curation, Formal analysis, Methodology, Resources, Visualization, Writing – original draft. KUs: Funding acquisition, Supervision, Writing – review & editing. VS: Supervision, Writing – review & editing. MM: Supervision, Writing – review & editing.

## Acknowledgements

The authors would like to acknowledge the land on which this research was undertaken, the ancestral lands of Gamilarraay, the lands, skies, and waters that are now known commonly as Australia, the importance of Country within Australian Indigenous concepts of wellbeing and resilience, the ongoing struggle for Indigenous survival linked to the wellbeing of Country, the role that decolonising research has in highlighting the importance of Indigenous perspectives and the members of the Aboriginal Community of Tamworth who have contributed to the formation and completion of this study, and the Australian Government's Department of Health and Aged Care. Additionally, we would like to acknowledge the contribution and continued support of the Lowitja Institute.
