# Supplementary material for: Understanding solastalgia from a decolonised, Indigenist lens: a scoping review
Source: Front Public Health. 2024 Jan 15;11:1261247. doi: 10.3389/fpubh.2023.1261247 (PMC10824238; doi:10.3389/fpubh.2023.1261247)
Supplement: Supplementary file 1 [file Data_Sheet_1.pdf]

Data Sheet 1: Methodologies and Frameworks

Circular/iterative Storytelling[1]

This paper has been written using a *circular* or *iterative* storytelling methodology, whereby, the development of this project is reflected in the writing style and structure of the paper. Additionally, the storytelling circle reflects the cultural and ethical protocols of Aboriginal research.[1] Therefore, the positioning of the story is acknowledged at the beginning and throughout the text. Story is an important part of maintaining Aboriginal Cultural Knowledge.

"Storytelling is a valid form of Aboriginal knowledge as it includes responsibility on the part of the listener/ researcher, incorporates both interpretation and analysis, has room for many explanations for the phenomena being researched, is a creative search for solutions, and is a political act of liberation and self-determination."[1]

Heuristic Inquiry (Indigenist)[2]

The conventional structure of heuristic inquiry was modified to include elements of personal positioning known as "Aboriginal ways of knowing, being and doing",[3] yarning (discussions) and creative translational artistic outputs. The Indigenist heuristic inquiry methodology is yet to be published. However, the method was informed by the methodology *Indigenous heuristic action research*, created by Kü Kahakalau.[2]

Aboriginal Participatory Action Research (APAR)[4]

Aboriginal participatory action research[4] is a critical research methodology. APAR differentiates from the general participatory action research framework by aligning with the guiding principles of Indigenous self-determination, empowerment and cultural recognition.[4] This methodology ensures that the project aligns with recommendations set by the National Health and Medical Research Council (NHMRC) ethical guidelines for conducting research with (and for) Indigenous Peoples.[5] As such, this paper was developed in conjunction with the local Aboriginal Community and as such, has recognised their contributions and acknowledged the Countries with which the knowledge has originated.

Yarning Methodology[6]

In addition to APAR, yarning is an Indigenous qualitative research methodology and was incorporated into the consultation process with the local Aboriginal Community. In this methodology, *yarning* (or discussions) are led by the researcher where the participant is encouraged to tell their story from the position of their lived experience.[6] This process was led by a series of informal questions, which allowed for open discussion about the topic.

Quandamooka' by Martin & Mirraboopa[3]

The Indigenist research framework called 'Quandamooka' by Martin & Mirraboopa[3] was consulted to ensure a culturally conscious and safe practice of this review:

For Indigenist research to be recognised by the western research academy it must also identify its methodology. But western research is a western practice and, as such, it is not a feature of our own world, so a research framework that is entirely Aboriginal is not possible. So Indigenist research occurs through centering

Aboriginal Ways of Knowing, Ways of Being and Ways of Doing in alignment with aspects of western qualitative research frameworks.[3]

The essential components of 'Quandamooka'[3] are first, to identify and position one's self. Secondly, acknowledge the origins of ones 'ways of knowing' or 'ontology', that being, the 'Entities of Land, Animals, Plants, Waterways, Skies, Climate and the Spiritual systems of Aboriginal groups'. Thirdly, identify one's 'ways of being', such as, our relationships to Country, human and non-human kin and that which is more-than-human. Fourthly, identify one's 'ways of doing', a way of synthesis, determined by one's 'ways of knowing and being'.

Martin & Marraboopa,[3] explain that "Indigenist research must centralise the core structures of Aboriginal ontology as a framework for research". By centering one's self within the research paradigm, a decolonised, Indigenist lens, is inherently infused into the synthesised framework. Therefore, by first performing the process of heuristic inquiry before conducting this review, KLU has inherently embedded both Gamiliarraay and personal ways of 'knowing, being and doing' in the conceptual understanding of the term solastalgia.

The Joanna Briggs Institute methodology for scoping reviews[7]

Outlined within Text.

PAGER framework[8]

Outlined within text.

EndNote[9] software

Outlined within text.

References

1. Baskin C. Storytelling circles: reflections of aboriginal protocols in research. Can
Soc Work Rev. (2005) 22:171–87.
2. Kahakalau K. Indigenous heuristic action research: bridging western and indigenous research methodologies. Multidiscipl Res Hawaiian WellBeing. (2004) 1:19–33.
3. Martin K, Mirraboopa B. Ways of knowing, being and doing: a theoretical framework and methods for indigenous and indigenist research. J Austral Stud. (2003) 76:203–14. doi: 10.1080/14443050309387838
4. Dudgeon P, Bray A, Darlaston-Jones D, Walker R. Aboriginal Participatory Action Research: An Indigenous Research Methodology Strengthening Decolonisation and Social and Emotional Wellbeing, Discussion Paper. Melbourne: Lowitja Institute (2020).
5. National Health and Medical Research Council. Ethical Conduct in Research with Aboriginal and Torres Strait Islander Peoples and communities: Guidelines for Researchers and Stakeholders. Canberra, ACT: NHMRC (2018).

6. Kennedy M, Maddox R, Booth K, Maidment S, Chamberlain C, Bessarab D. Decolonising qualitative research with respectful, reciprocal, and responsible research practice: a narrative review of the application of Yarning method in qualitative Aboriginal and Torres Strait Islander health research. Int J Equity Health. (2022) 21:134. doi: 10.1186/s12939-022-01738-w
7. Bradbury-Jones C, Aveyard H, Herber OR, Isham L, Taylor J, O'Malley L. Scoping reviews: the pager framework for improving the quality of reporting. Int J Soc Res Methodol. (2022) 25:457–70. doi: 10.1080/13645579.2021.1899596
8. Bradbury-Jones C, Aveyard H, Herber OR, Isham L, Taylor J, O'Malley L. Scoping reviews: the pager framework for improving the quality of reporting. Int J Soc Res Methodol. (2022) 25:457–70. doi: 10.1080/13645579.2021.1899596
9. The EndNote Team. EndNote (EndNote X9). Philadelphia, PA: Clarivate (2013).
