# Supplementary material for: Understanding solastalgia from a decolonised, Indigenist lens: a scoping review
Source: Front Public Health. 2024 Jan 15;11:1261247. doi: 10.3389/fpubh.2023.1261247 (PMC10824238; doi:10.3389/fpubh.2023.1261247)
Supplement: Supplementary file 2 [file Data_Sheet_2.pdf]

Data Sheet 2: Most Common Words

Word	Count	Similar Words
Changing	481	change, changed, changes, changing
Climatic	398	climate, climatic
Humans	359	human, humanism, humanities, humanity, humans
Community	306	communicate, communicated, communicates, communicating, communication, communications, communicative, communities, community
Country	267	countries, country
Ways	249	way, ways
People	245	people, peoples
Becoming	233	become, becomes, becoming, becomings
Placing	228	place, placed, places, placing
Indigenous	217	Indigenous
Research	191	research, researched, researcher, researchers, researching
Adaptive	189	adapt, adaptability, adaptation, adaptations, adapted, adapting, adaption, adaptive
Relations	183	relate, related, relates, relating, relation, relational, relationality, relationally, relations, relative, relatively, relatives
Time	183	time, times, timing
Understandings	168	understand, understandably, understanding, understandings
Bawaka	152	bawaka
Participants	150	participant, participants, participate, participated, participation
Knowledge	141	knowledge, knowledgeable, knowledges
Responsiveness	132	response, responses, responsibilities, responsibility, responsible, responsive, responsively, responsiveness
Knowing	129	know, knowing, knowings, knows
Including	124	include, included, includes, including
Relationships	121	relationship, relationships
Space	121	space, spaces
Many	120	many
Differently	116	differ, difference, differences, different, differently, differing, differs
Connects	112	connect, connected, connecting, connection, connections, connectivity, connects
Caring	107	care, cared, careful, carefully, cares, caring
Discussions	107	discuss, discussed, discussing, discussion, discussions
Important	105	importance, important, importantly
Weather	105	weather, weathering, weathering
Lands	102	land, lands
May	100	may
Cultures	99	cultural, culturally, culture, cultures
Local	97	local, localities, locally
World	92	world, worlds
Socially	91	social, sociality, socially
Future	90	future, futures
Particular	89	particular, particularly
Sharing	88	share, shared, shares, sharing
