# Supplementary material for: Understanding solastalgia from a decolonised, Indigenist lens: a scoping review
Source: Front Public Health. 2024 Jan 15;11:1261247. doi: 10.3389/fpubh.2023.1261247 (PMC10824238; doi:10.3389/fpubh.2023.1261247)
Supplement: Supplementary file 3 [file Data_Sheet_3.pdf]

**Data Sheet 3.** Data Extraction Table.

| Author, Year of Publication and Country | Aim/Purpose | Methodology/ Methods | Key Findings | Connection to Country | Knowledge translation/ traditional knowledge & practices | Climate Change | Adaption | Emerging Themes |
|---|---|---|---|---|---|---|---|---|
| Petheram, L., Zander, K. K., Campbell, B. M., High, C., & Stacey, N. (2010). 'Strange changes': Indigenous perspectives of climate change and adaptation in NE Arnhem Land. Global Environmental Change. 20(4). | The aim of this study is to "improve understanding of factors important in integration of Yolngu perspectives in planning adaptation policy in North East Arnhem Land (Australia)" (pp.1).<br><br>Data analysis aimed to "provide insights into participants' perspectives on their environment, living situation and climate change" (pp.15). | -Glaser's (1992) grounded theory methodology (pp.12)<br><br>-Relationship building/fieldwork between 2008-2010<br><br>-Workshops<br><br>-Semi-structured interviews<br><br>-'rich picture diagramming' (RPD)<br><br>-'participatory sculpting'<br><br>-'participant generated photography'<br><br>-Qualitative | -Participants ranged in age and gender<br><br>-"Yolngu people have different roles, responsibilities and laws regulating the type of knowledge they are able to share, especially to those outside their clan" (pp.12)<br><br>-Location described as- Quote: "'a common location occupied by the traditional owners or those people with a direct link to the traditional owner of the land...and may or may not be permanently occupied throughout the year" (pp.7)<br><br>-Differing perspective of colonial intervention<br><br>-Many Yolngu communities continue a nomadic lifestyle<br><br>-Participants were more familiar with traditional languages, rather than English<br><br>-Central concept identified as 'current vulnerability'<br><br>-Expressing desire for "greater respect and decision-making power to be given to Yolngu, and for the community freedom, empowerment, independence and action" (pp.18)<br><br>-Lack of local services<br><br>-Lack of empowerment and voice<br><br>-'Climate change', an unfamiliar topic, general understanding | - ALL participants "exhibited strong connection to place, and sensitivity to natural landscape." (pp.16)<br><br>-Interconnection to Country<br><br>-Emphasis on maintaining and continuing traditional practices and the passing of knowledge<br><br>-Expression of 'enjoyment' in taking care of Country<br><br>-"deep connections with 'country' and the way that the condition of their country affects their cultural, mental and physical Health" (pp.16)<br><br>-Discussion of the negative environmental impact of a nearby mine and how it "affected them to their core"( pp.16)<br><br>-'hurt and frustration' over the mistreatment and mismanagement of Country<br>- Description of miscommunication between communities and government. | -" participants were very generous in sharing personal (traditional and contemporary) songs, stories, chanting, dancing and art during engagement with our non-Indigenous research team" (pp.18)<br><br>-Pride in creative expression and Culture<br><br>-Expressing a need to be free to use traditional knowledge and practices | -Informed participants expressed a concern over the increasing likelihood of worsening negative effects<br><br>-Participants linked climate change effects to worsening medical issues<br><br>-changing landscape described as "strange changes" (pp.21)<br><br>-Participants 'unsure' of the cause of climate change, but associated the cause with human intervention- "big cities" (pp.21)<br><br>-Quote: "what we are doing to mother nature. Mother nature is now weeping" (pp.21)<br><br>-Mention of 'spirits' being involved, descriptions of 'feeling' the shift in Country<br><br>-Landscape changes related to increasing recreation and tourism<br><br>-Decreasing number of traditional plants and animals observed<br><br>-Discussions of negative mental and physical health linked to "impaired health of the natural landscape" (pp.22)<br><br>-Quote: "the altered distribution and abundance of animal and plant species would markedly affect hunting and other cultural practices, and exacerbate current health problems" (pp.23) | -Maintaining traditional practices as a way of "staying strong" (pp.23)<br><br>-Continuing traditional lifestyle imperative<br><br>-Living on and connecting to Country is essential<br><br>-Quote: "Participants stressed the importance of dealing with problems in culturally acceptable ways" (pp.23)<br><br>-Natural food and resources, important to good health<br><br>-Potential of using 'western' knowledge to adapt to a changing environment<br><br>-Kinship support and connection is "essential to community survival" (pp.23)<br><br>-Collaborative processes of designing and implementing services, essential | Recognition of Knowledge derived from Country<br><br>Aboriginal Collaboration<br><br>Storytelling Methodologies<br><br>Importance of Traditional Language<br><br>Aboriginal Perspectives & Experience<br><br>Connection to Country & links to Environmental Changes<br><br>Adaption<br><br>Interconnection of Humans & Country |

| | | | | | | | | |
|---|---|---|---|---|---|---|---|---|
| Bawaka Country, Wright, S., Suchet-Pearson, S., Lloyd, K., Burarrwanga, L., Ganambarr, R., Ganambarr-Stubbs, M., Ganambarr, B., & Maymuru, D. (2020). Gathering of the Clouds: attending to Indigenous understandings of time and climate through songspirals. Geoforum. (108). | The aim of this paper is to educate and share knowledge and create an understanding of time and climate change led by the Yolŋu songspiral, gathering of the clouds (Wukun). | - Bawaka Country, Indigenous and non-Indigenous authors, share their collective understanding of Yolŋu philosophical understandings and knowledge<br><br>-Translational methods of story, song and understandings | -Climate change is not separate to the human experience<br><br>-Modern social constructs of measurement, time and the separation of humans to the environment, has led to a desensitised perspective of environmental change and destruction<br><br>-The human impact has been separated from the environmental impact of climate change<br><br>-This paper is led by Country through the discussion of songspirals/songlines<br><br>-This discussion paper took 12 years of learning in order to translate into words<br><br>-The past, present and future are one | -There is no separation between human and non-human. " The clouds are both people and Country and the songspiral makes and re-makes both" (pp.296)<br><br>-Country is embodied in songspirals/songlines<br><br>-Collaborative co-becoming with Country<br><br>-Singing songlines is a traditional method of connecting and co-becoming Country, a practice that is spiritual and physical | -Traditional systems knowledges are translated into an accessible comprehensible format<br><br>-The use of story as methodology as a translation technique is effective<br><br>-Quote: "Our collaboration is itself a process of co-becoming as Yolŋu, ŋapaki and non-human beings co-constitute each other through relationships which are always in emergence". (pp. 296) | -Quote:" climate is often defined in a technocratic manner, abstracted, linear and measured" (pp.295)<br><br>-With regards to climate change, "There is little recognition of the deep material and symbolic connections that enable cultures, beings, becomings, and places" (pp.295)<br><br>-The future cannot be predicted or controlled | -Quote: "The rain begins. The nourishing rain that revives and makes anew. It rains the trees, the tracks, the plants, all that is Country… And when it rains, that land cools down and that is the tears; it stills our grief and sorrow and we return home". (pp.302) | Recognition of Knowledge derived from Country<br><br>Aboriginal Collaboration<br><br>Storytelling Methodologies<br><br>Importance of Traditional Language<br><br>Aboriginal Perspectives & Experience<br><br>Connection to Country & links to Environmental Changes<br><br>Adaption<br><br>Interconnection of Humans & Country |
| Nash, D., Memmott, P., Reser, J., & Suliman, S. (2018). We're the same as the Inuit!: exploring Australian Aboriginal perceptions of climate change in a multidisciplinary mixed methods study. (Australia) | -Firstly, "explore the perceptions, understandings, and responses to media coverage and possible local experiences of perceived climate change of Aboriginal people in the Upper Georgina River Basin". (pp.108)<br><br>-Secondly, "study the adaptive capacity of these communities in the face of the perceived threats of climate change, and to broader environmental impacts, particularly with regard to emergency and disaster management, land and riverine management, housing and settlement adaptation, and enterprise development responses". (pp. 108) | -APAR<br><br>-Qualitative RM<br><br>-Quantitative RM | -A modified survey, as used within this study, was proven to reflect more accurate results. Suggesting that the implementation of this strategy was improved<br><br>-Quote: "Indigenous worldviews were influencing the respondents' attitudes and knowledge of environmental changes, including identifying these changes as a cause for 'worry'". (pp.113)<br><br>-Survey results were used to inform the content discussed within the subsequent workshops | -Quote: "often-mentioned cultural and regional difference is that these communities' connection to their traditional 'country' will make any recommended relocations in the face of climate change impacts extremely difficult". (pp.117) | -Identification of the understandings of both western and Traditional sciences are important | -Quote: "Aboriginal risk perceptions and understandings of climate change are both important and changing—important because the deep connection to the land provides important insights into the ways that climate change affects local environments, and changing because of the changing relationships between Aboriginal people, traditional knowledge systems, and popular understandings of climate change phenomena". (pp.117) | -Suggestions of collaborative adaption strategies and policies | Aboriginal Collaboration<br><br>Storytelling Methodologies<br><br>Aboriginal Perspectives & Experience<br><br>Connection to Country & links to Environmental Changes<br><br>Adaption<br><br>Interconnection of Humans & Country |

| | | | | | | | | |
|---|---|---|---|---|---|---|---|---|
| Petheram, L., Stacey, N., & Fleming, A. (2015) Future sea changes: Indigenous women's preferences for adaptation to climate change on South Goulburn Island, Northern Territory. (Australia) | -Develop an understanding of Aboriginal perspectives of climate change<br><br>-Promote community awareness of climate change phenomena and resulting potential increasing threat and impact | -Qualitative RM<br><br>-Adaptive RM | -Findings suggest that participants are aware of what climate change is, however, they did not relate it specifically to their own observations of a changed environment | -Quote: "participants felt a very intuitive connection to the natural environment – and gave strong agency to many elements in the land/seascape, such as their close relationships with plants and animals". (pp.346) | -Some participants identified that English was not their primary language<br><br>-Stories not often featured in their entirety | -workshops doubled as an education session of the impact of climate change<br><br>-Quote: "Western concepts of climate change were quite unfamiliar to most local residents on South Goulburn Island, prior to the discussions in this research". (pp.344) | -Quote: "participants preferred to discuss climate change concepts and potential adaptations in the context of other (higher priority) community issues and aspirations". (pp.347) | Aboriginal Collaboration<br><br>Storytelling Methodologies<br><br>Aboriginal Perspectives & Experience<br><br>Connection to Country & links to Environmental Changes<br><br>Adaption<br><br>Interconnection of Humans & Country |
| Bawaka Country., Wright, S., Suchet-Pearson, S., Lloyd, K., Burarrwanga, L., Ganambarr, R., Ganambarr-Stubbs, M., Ganambarr, B., Maymuru, D., & Sweeney, J. (2016). Co-becoming Bawaka: Towards a relational understanding of place/space. (Australia) | -To use story as a way of translating the practice of co-becoming Country<br><br>-Quote: "Part of what we aim to do in attending to Bawaka as a co-author is to respect and acknowledge both our co-constitution and the active role that Country plays in shaping what we know, who we are and what we do". (pp.463) | -Storytelling<br><br>-Traditional knowledge translation | - Perspective piece<br><br>-Quote: "This paper would not have been written the way it is, would not include the words it does, if Yuwululsurra had not brought ganguri back from Burralku." (pp.468) | -Quote: "Yes, the academics in the research collective reshaped our words for the paper, submitted it and responded to the reviewers and editors. However, this paper was authored by Bawaka Country." (pp.468) | -Quote: "Bawaka Country, as lead author of this paper, guided, inspired and enabled. It empowered learning, opened avenues of discussion and made us – human and more-than-human – who we are. The experiences, understandings and learnings this paper contains would not exist if not for Bawaka Country, for Rom, which underlies it and gurrutu, which shapes it. And as you will now realize, this fundamentally includes its nine human co-authors who co-become with and as a part of Bawaka – not indistinguishable from it, but continually shaped by their intrarelationships." (pp.468-469) | -Ignoring the more-than-human in the realm of climate change, potentially through 'greater than' thinking, creates a disconnected way of thinking and perceiving the issue of climate change. | -The word 'adapt' is not found within this paper.<br><br>-This paper wishes to highlight relatability, and the importance of understanding our relationship to Country, rather than separating the Human from the Earth. | Recognition of Knowledge derived from Country<br><br>Aboriginal Collaboration<br><br>Storytelling Methodologies<br><br>Importance of Traditional Language<br><br>Connection to Country & links to Environmental Changes<br><br>Interconnection of Humans & Country |
| Bawaka Country as Suchet-Pearson, S., Wright, S., Lloyd, K., Burarrwanga, L. and (2013), Caring as Country. (Australia) | -Storytelling to highlight the importance of Caring AS Country, rather than Caring FOR Country. | -Storytelling- Yolŋu traditional story of gathering miyapunu mapu (turtle eggs). | -This story represents the act of using, maintaining and caring for Natural Resources.<br><br>-Specifically states- NRM- Natural Resource Management<br><br>-Mis-managed Natural Resources lead to dire consequences for Country and Community. | -Yolŋu ontology of 'co-becoming' Country.<br><br>-Co-becoming is a function of NRM. | -Storytelling<br><br>- Yolŋu Ontology<br><br>-Traditional methods of NRM<br><br>-Battle between Yolŋu and outside agencies, to continue Caring AS Country. | -Fighting for rights to continue traditional practices.<br><br>-Quote: "Indigenous peoples and local communities have profoundly challenged the dominance of industrial resource management regimes in a variety of | -Relationships<br><br>-Working together with outside agencies<br><br>-Collaboratively looking after Country with Non-Indigenous Peoples | Recognition of Knowledge derived from Country<br><br>Aboriginal Collaboration<br><br>Storytelling Methodologies |

| | | | | | | | | |
|---|---|---|---|---|---|---|---|---|
| | | | | | | ways over many centuries.” (pp.188) | | Importance of Traditional Language<br><br>Connection to Country & links to Environmental Changes<br><br>Interconnection of Humans & Country |
| McNamara, K., & Westoby, R. (2011) Solastalgia and the Gendered Nature of Climate Change: An Example from Erub Island.<br>(Torres Strait) | -Quote: “explore the gendered nature of climate change, and provide new perspectives on how these environmental impacts are experienced, enacted and responded to”. (pp.233) | -Semi-structured interviews<br><br>-Semi-conducted literature review and research project | -This paper states that the narratives as gifted by the Elders of Erub Island, conclusively align with the concept of solastalgia | -Results depict a decreasing familiarity to land and deteriorating connection to both self and Country | -Stories were not featured in their entirety<br><br>-Stories by ‘Aunties’- gendered nature- Matriarchs of the Community | -States “To date, little published work examines the gendered nature of climate change.” (pp.234)<br><br>-‘Indigenist Lens’ identified for climate change impact- storytelling.<br><br>-Quote: “The insights of these Aunties can provide novel perspectives on how climate and environmental change impacts are interpreted, experienced and reacted to.” (pp.235) | -Exploring climate change resilience and adaptive practices<br><br>-Encourages outside agencies to work with Indigenous Peoples to mitigate and adapt to climate change. | Aboriginal Collaboration<br><br>Storytelling Methodologies<br><br>Aboriginal Perspectives & Experience<br><br>Connection to Country & links to Environmental Changes<br><br>Adaption |
